# Will Next Match Location Influence External and Internal Training Load of a Top-Class Elite Professional European Soccer Team?

**DOI:** 10.3390/ijerph18105229

**Published:** 2021-05-14

**Authors:** Rafael Oliveira, João Paulo Brito, Nuno Loureiro, Vítor Padinha, Hadi Nobari, Bruno Mendes

**Affiliations:** 1 Sports Science School of Rio Maior–Polytechnic Institute of Santarém, 2040-413 Rio Maior, Portugal; jbrito@esdrm.ipsantarem.pt (J.P.B.); nunoloureiro@esdrm.ipsantarem.pt (N.L.); vitorpadinha@esdrm.ipsantarem.pt (V.P.); 2 Life Quality Research Centre, 2040-413 Rio Maior, Portugal; 3 Research Centre in Sport Sciences, Health Sciences and Human Development, 5001-801 Vila Real, Portugal; 4 Department of Physical Education and Sports, University of Granada, 18010 Granada, Spain; hadi.nobari1@gmail.com; 5 Department of Exercise Physiology, Faculty of Sport Sciences, University of Isfahan, Isfahan 81746-7344, Iran; 6 HEME Research Group, Faculty of Sport Sciences, University of Extremadura, 10003 Cáceres, Spain; 7 Sports Scientist, Sepahan Football Club, Isfahan 81887-78473, Iran; 8 Faculty of Human Kinetics, University of Lisboa, 1649-002 Lisboa, Portugal; brunomendes94@hotmail.com

**Keywords:** soccer training, s-RPE, Hooper index, GPS, match day, match location

## Abstract

Background: The purpose of this study is to compare training load (TL) preceding a home versus away match in a top-class elite European team during the 2015–2016 season. Methods: Twenty elite outfield soccer players with a mean ± SD age, height and body mass of 25.9 ± 4.6 years, 183.1 ± 6.6 cm and 78.6 ± 6.6 kg, respectively, participated in this study. Total distance covered, high-speed running distance (HSRD), average speed (AvS), rating of perceived exertion (RPE) multiplied by training duration (s-RPE) and Hooper index (HI) were collected. Data from 24 weeks were analyzed through match-day minus/plus approach (MD-5, -4, -3, -2, -1, MD + 1). Results: All external TL variables indicated a decrease from MD-5 until MD-1 and then an increase to MD + 1 (*p* < 0.01). HI decreased from MD-5 to MD-1, but s-RPE increased until MD-3 and then decreased until MD + 1. When comparing TL data that preceded home matches versus away matches, for MD-5, HSRD and muscle soreness exhibited higher values when away match neared (*p* < 0.05). For MD-4 and MD-3, total distance, HSRD and AvS exhibited higher values closer to an away match than a home match (*p* < 0.05). For MD-1, total distances covered were higher closer to a home match than an away match (*p* < 0.01). For MD + 1, all HI items and AvS were higher when an away match was played (*p* < 0.05). Conclusions: This study confirms and provides evidence regarding the influence on internal and external TL data preceding home and away matches from a team that played in European competitions.

## 1. Introduction

Several situational variables (e.g., competition stage, match location, quality of opposition and match status or match outcome) impact a sports team’s performance [1]. Soccer is dominated by strategic/tactical factors; therefore, it is reasonable to suggest that situational variables influence team and player performance [2,3,4,5,6]. Match location (playing home or away) has been identified as one of the most important situational variables that dictate possession patterns [7,8]. Playing a home match implies stronger interaction with team possession than playing an away match [4,6,9]. In addition, other studies have revealed that indicators such as stress and sleep are influenced by match location [10,11].

Furthermore, soccer science has begun to focus more on delimiting success indicators in soccer. Nevertheless, soccer science in general is inconclusive due to small sample size, unstandardized analysis procedures and lack of consideration of the complexity of soccer as an unpredictable and dynamic sport. Even so, there are useful variables for quantifying, modeling and possibly adjusting internal and external training load (TL). One way to control internal TL involves multiplying the duration of training sessions by the rating of perceived exertion (s-RPE), also known as training impulse [12,13,14,15,16,17]. Another way to control internal TL is the wellbeing status provided by the Hooper index (HI) questionnaire that measures the perception of fatigue, stress, delayed onset muscle soreness (DOMS) and sleep quality [18,19].

Recently, both methods were used to control internal TL [19,20,21,22]. Nobari et al. [19] found highest values of weekly acute, chronic and strain s-RPE were verified in the mid-season and the lowest values in the early season; the highest values of accumulated weekly fatigue, stress, and DOMS were observed in the late season, and the lowest values of sleep and stress were found in the early season, while the lowest values of fatigue and DOMS were observed in the mid-season. Clemente et al. [21] found that the relationship between s-RPE and HI indicates significant and negative small-to-moderate correlations in the weeks with two matches, but not in the weeks with only one match. On the other hand, Oliveira et al. [22] showed minor variations regarding HI scores across 10 mesocycles and in days prior to the match. In fact, only the day following the match revealed increases in HI scores. This last finding was also corroborated by Oliveira et al. [20]. 

Based on a previous study, a combined analysis of contextual effects on TL, determined by s-RPE, different running distances and wellbeing status, accumulated within a match-to-match microcycle, can provide more rounded information to improve understanding of the demands of match play [20].

To the best of our knowledge, only two studies about training content have attempted to assess the difference between playing a home or an away soccer match for internal TL of elite professional European soccer teams [11,23]. Abbott et al. [11] analyzed the influence of these situational match variables on subjective wellbeing status (fatigue, DOMS, quality sleep, stress and mood) in under-23 soccer players after several matches throughout a season of English Premier League 2 and found that subjective wellbeing does not differ before the match (*p* > 0.05). However, on the first and third days following the match, stress and mood were ≥20% lower after playing away from home (*p* < 0.05). Meanwhile, Brito et al. [23] analyzed situational variables of subjective wellbeing in under-19 players from a first league club in France and found that subjective wellbeing was not affected by match location. However, subjective wellbeing was only assessed the day before the match, and as the authors acknowledged, this might not be the most suitable time to assess the influence of these variables on match-to-match fluctuations in wellbeing. Thus, the present study is the first to explore whether match location affects the training sessions that precede a home or away match (regarding internal and external TL variables) in a team that plays European competitions through the full season. 

Therefore, the main purpose of this study is to compare internal and external TL preceding a home versus away match. A secondary purpose was to determine if there were differences between starters and non-starters in a top-class elite European team during the 2015–2016 season.

## 2. Materials and Methods

### 2.1. Experimental Approach to the Problem

TL data were collected over 39 weeks during the 2015–2016 season. All elite players competed in four official competitions throughout the season, including the European Competition, the national league and two national cups from their own country. For the purposes of the present study, only main team sessions were considered. Only data from the training sessions were considered. Data from rehabilitation or recuperation were excluded. The total minutes of each training session comprised a warm-up, a main phase and a slow-down phase, in addition to stretching. Data that preceded 12 home matches and 12 away matches were analyzed. All matches were national league matches. Training data collection for this study was carried out at the soccer club’s outdoor training pitches. To analyze data, only weeks with one match were included, and the approach in relation to the number of days away from the competitive match fixture (i.e., match day minus (MD-)) was used [20,22]. The team typically trained five days per week (MD-5, MD-4, MD-3, MD-2, MD-1), plus one day after the match (MD + 1). Due to the different week schedules, there were some weeks with a day off on MD + 1, and for that reason, in some weeks, MD-5 was the first training day of the week. The number of MD minus/plus is identified in the results section (Table 1). 

### 2.2. Participants

The sample consisted of four central defenders (CDs), four wide defenders (WDs), five central midfielders (CMs), four wide midfielders (WMs) and three strikers (ST) of an elite European soccer team that plays in the UEFA Champions League. The players exhibited a mean ± SD age, height and mass of 25.85 ± 4.55 years, 183.06 ± 6.64 cm and 78.56 ± 6.64 kg, respectively. Height and weight were collected through a scale and stadiometer (SECA 220, Germany, Hamburg) to the nearest 0.01 kg and 0.1 cm, respectively. Inclusion criteria are described by Oliveira et al. [20] and mean that participants had regular participation with a minimum of 80% weekly training sessions. Participants also had to complete at least 60 min in one match in the first half of the season and one match in the second half of the season. For further analysis, we added other inclusion criteria to analyze MD + 1 and MD-5 by dividing starters and non-starters. Players were considered starters if they participated in three consecutive matches for at least 60 min, while the other players were considered non-starters [20]. All participants were familiarized with the training protocols and signed informed consent prior to the investigation. This study was conducted according to the requirements of the Declaration of Helsinki and was approved by the Ethics Committee of Polytechnic Institute of Santarém (252020Desporto). 

### 2.3. External Training Load—Training Data

Each player’s physical activity during each training session was monitored using portable global positioning system (GPS) units (Viper pod 2, STATSports, Belfast, UK). This device provides position velocity and distance data at 10 Hz frequency. It was used across the upper back between the left and right scapula through a custom-made vest that allows a better satellite reception for the GPS antenna. This system has previously been determined to be valid and reliable to measure linear, multidirectional and soccer-specific activities [24]. Thirty minutes before use, all devices were turned on in order to acquire satellite signals and to provide synchronization between the GPS clock and the satellite’s atomic clock [21]. After data collection, the Viper PSA software (STATSports, Belfast, UK) was used to download data and to clip the entire training session (i.e., from the beginning of the warm-up to the end of the last organized drill). Players wore the same GPS device for each training session to avoid interunit error. The following variables were assessed: total duration of training session (minutes), total distance and high-speed running distance (HSRD, above 19 km/h).

### 2.4. Internal Training Load—Training Data

The perceptions of fatigue, stress, DOMS and quality of sleep were assessed through the HI [18] 30 min before the beginning of training sessions. The scale of HI uses 1–7 points, in which 1 is very, very low and 7 is very, very high (for stress, fatigue and muscle soreness levels) and 1 is very, very bad and 7 is very, very good (for sleep quality). Then, the summation of the four categories provides the total HI. In addition, RPE, on a scale of 0–10 [25] was collected 30 min after the end of the training session. Then, it was multiplied by the session duration to generate a session RPE (s-RPE) [12,13,14,15,16,17]. 

### 2.5. Statistical Analysis

The SPSS version 22.0 (SPSS Inc., Chicago, IL, USA) for Windows statistical software package was used to analyze the data. To describe and characterize the sample, descriptive statistics were used. Shapiro–Wilk and Mauchly’s tests were performed to determine normality and sphericity, respectively. Once the variables reached normal distribution, repeated-measures ANOVA with a Bonferroni post hoc was used (Shapiro–Wilk > 0.05) to compare the days prior to the competitive match, as well as match location. ANOVA Friedman and Mann–Whitney tests were used for the variables for which normal distribution had not been obtained to compare different moments and different player positions. Independent sample *t*-test was used to compare data from starters and non-starters. Results were significant in the interaction (*p* ≤ 0.05). The Cohen’s d effect-size (ES) statistic was calculated to determine the magnitude of effects by the difference of two population means which are then divided by the standard deviation from the data, and it was assessed using the following criteria: <0.2 = trivial, 0.2–0.6 = small, 0.6–1.2 = moderate, 1.2–2.0 = large, and >2.0 = very large effect [26].

## 3. Results

We analyzed physical performance in the weeks that preceded the 24 analyzed matches (12 home and 12 away matches over the entire season). 

**Figure 1:**
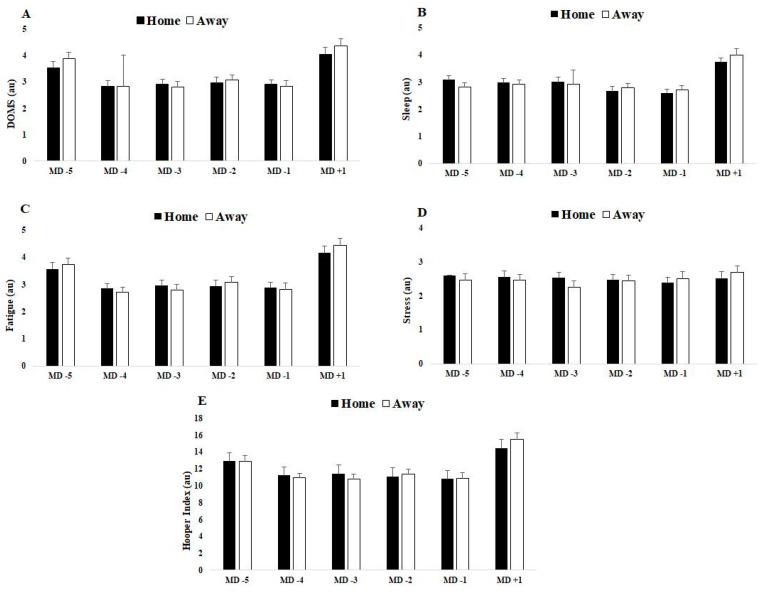
Differences between match day minus/plus preceding home and away matches for (**A**) DOMS, (**B**) sleep, (**C**) fatigue, (**D**) stress and (**E**) Hooper index. Data presented by mean ± SD.

**Figure 2:**
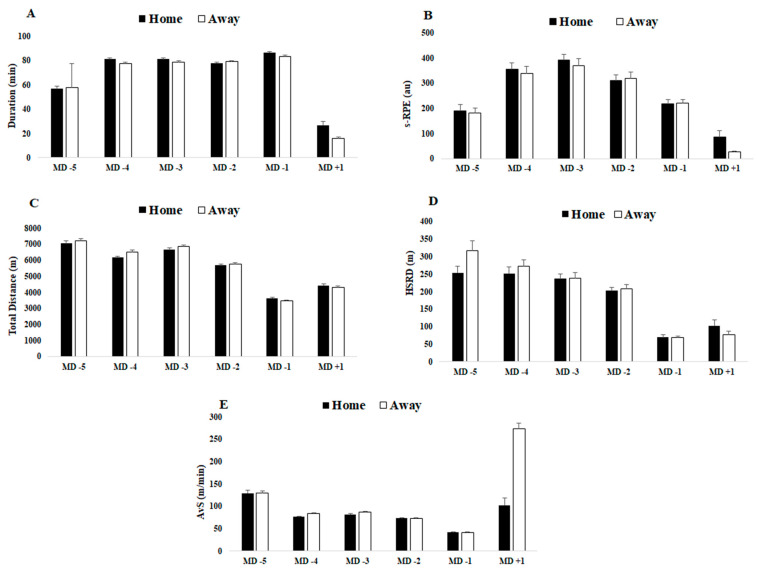
Differences between match day minus/plus preceding home and away matches for (**A**) duration, (**B**) s-RPE, (**C**) total distance, (**D**) HSRD and (**E**) AvS. Data presented by mean ± SD.

Descriptive results and comparisons of match day minus for TL data that preceded home or away matches and comparisons of TL data that preceded home versus away matches for squad average are presented in Table 1. Figure 1 and Figure 2 displayed a graphical representation of Table 1 through mean and standard deviation (SD).

### 3.1. Comparison of Match Day Minus Preceding Home or Away Matches

In general, and regardless of match location, based on internal TL data that preceded home and away matches, all categories from HI and the total HI scores were higher on MD + 1 than all of MD-(5, 4, 3, 2, 1), and the scores decreased from MD-5 to MD-1. Moreover, s-RPE values increased until MD-3 and then decreased until MD + 1. External TL total distance, HSRD and AvS values decreased from MD-5 to MD-1 and then increased to MD + 1. 

For data preceding home matches, the main results indicate that stress (from HI questionnaire) does not differ for all MD-(5, 4, 3, 2, 1) or for MD + 1. For MD-5 vs. MD-1, all variables exhibited differences, with the exception of s-RPE. When comparing MD-5 vs. MD + 1, sleep, HI (total), duration, s-RPE, total distance, HSRD and AvS exhibited differences. When comparing MD-4 vs. MD-1, all variables were different with the exceptions of DOMS, sleep and fatigue. When comparing MD-4 vs. MD + 1, all variables were different, except for total HI score. When comparing MD-3 and MD-2 vs. MD-1, all variables exhibited differences, except for every category of HI scores. Moreover, when comparing MD-3 and MD-2 vs. MD + 1, all variables differed, except for AvS. Lastly, when comparing MD-1 vs. MD + 1, all variables except HSRD exhibited differences. 

For data preceding away matches, the main results indicate that stress does not differ for any of the MD-(5, 4, 2, 1) but differs between MD-3 and MD + 1. When comparing MD-5 vs. MD-1, all variables were different, except sleep. When comparing MD-5 vs. MD + 1, all variables exhibited differences, except for DOMS. When comparing MD-4 vs. MD-1, only s-RPE, total distance, HSRD and AvS differed. When comparing MD-4 vs. MD + 1, all variables were different, except stress. When comparing MD-3 vs. MD-1, all variables exhibited differences. When comparing MD-3 vs. MD + 1, only s-RPE, total distance, HSRD and AvS differed. When comparing MD-2 vs. MD + 1, all variables differed, except for stress. When comparing MD-2 vs. MD + 1, only s-RPE, total distance, HSRD and AvS differed. When comparing MD-1 vs. MD + 1, all variables exhibited differences, except stress and HSDR. 

Finally, Table 2 presents differences between starters vs. non-starters regarding MD + 1 and MD-5. Only data regarding home matches presented significant differences in DOMS (*p* = 0.018), stress (*p* = 0.030) and HI (*p* = 0.030) for MD + 1.

### 3.2. Comparison of Match Location 

When comparing TL data that preceded home matches versus away matches, for MD-5, it was observed that total distance, HSRD and DOMS from the HI questionnaire exhibited higher values, while s-RPE was lower when closer to an away match than a home match. 

For MD-4 and MD-3, external TL variables, such as total distance, HSRD and AvS, exhibited higher values when an away match was nearer, although all internal TL variables exhibited higher values when a home match was nearer.

For MD-2, all external and internal TL variables presented higher values closer to an away match than a home match, except for stress.

For MD-1, duration of training sessions and total distance covered were higher closer to a home match than an away match.

For MD + 1, all scores from the HI questionnaire and AvS were higher after playing an away match than a home match, although the duration of the training sessions, s-RPE, total distance and HSRD were lower after playing an away match than a home match.

**Figure 3:**
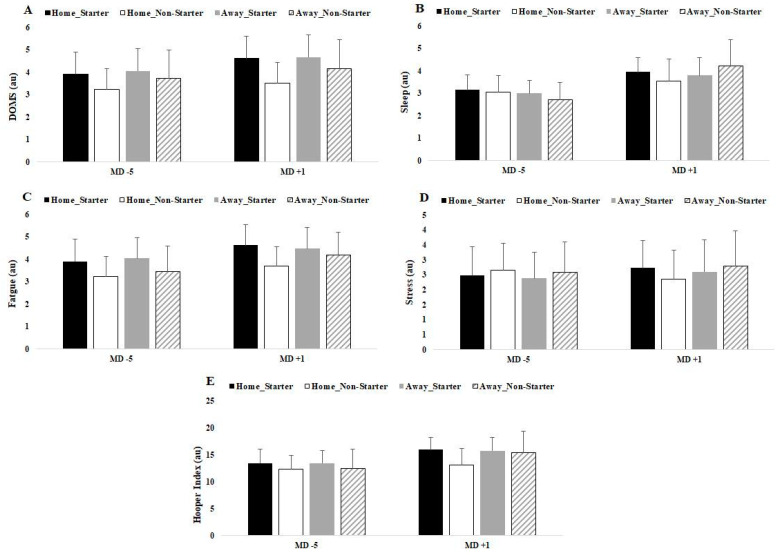
Differences between match-day minus/plus preceding home and away matches by player positions for (**A**) DOMS, (**B**) sleep, (**C**) fatigue, (**D**) stress and (**E**) Hooper index. Data presented by mean ± SD.

**Figure 4:**
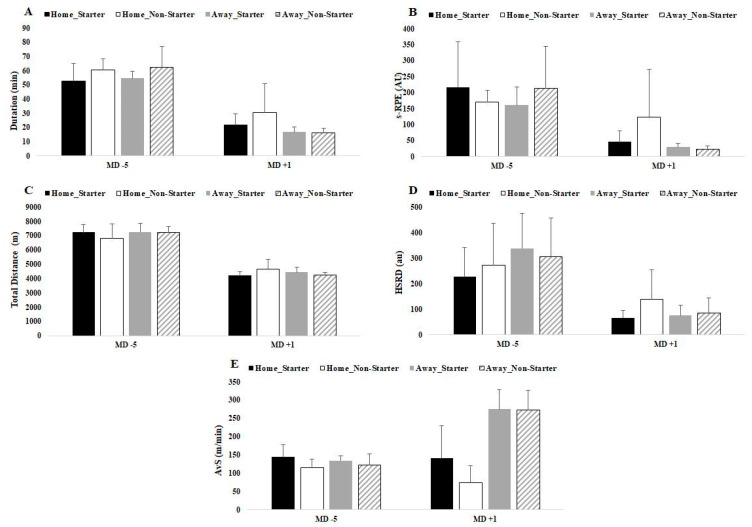
Differences between match day minus/plus preceding home and away matches for (**A**) duration, (**B**) s-RPE, (**C**) total distance, (**D**) HSRD and (**E**) AvS. Data presented by mean ± SD.

Figure 3 and Figure 4 displayed a graphical representation of Table 2 through mean and SD.

## 4. Discussion

The purpose of the present study was to quantify the internal and external TL employed by a top European soccer team during a full season to compare match day minus for TL that precedes a home or away match, as well as compare TL that precedes a home match versus TL that precedes an away match.

### 4.1. Comparison of Match Day Minus Preceding Home or Away Matches

In general, the first finding is a decrease for all variables on MD-1, which is in accordance with several other studies [27,28,29]. 

For internal TL, s-RPE revealed similar results regardless of match location when compared to other studies that found a progressive increase in this variable until MD-3, with a subsequent decrease until MD-1 [27,28,29]. Regarding MD + 1, s-RPE was higher than on MD-1 but lower than on MD-(5, 4, 3, 2) as home matches neared, while s-RPE was lower than on all MD-(5, 4, 3, 2, 1) for away matches. These results can be attributed to the recovery training sessions that occurred the day following the match. 

In addition, HI scores revealed few variations in the days prior to each match, with the highest values being reported on the day after a match, which supports the claim that matches represent the most demanding workload of each week [28]. Moreover, MD-5 had higher values for DOMS, fatigue, sleep and total HI than MD-(4, 3, 2, 1), although these MD-(4, 3, 2, 1) are similar when home or away matches approach all variables. Moreover, these results are in accordance with those reported by Haddad et al. [30], who suggested that fatigue, stress, DOMS and sleep are not major contributors to perceived exertion during traditional soccer training without excessive TL. However, these findings also oppose the results of Clemente et al. [19,21], who found that the relationship between s-RPE and HI is both significant and negative in the weeks that contained two official matches, but not in the weeks with only one match. 

Furthermore, for external TL variables, our study reveals that MD-5 has the highest TL session with lower duration and that TL was successively reduced until MD-1 even with higher training durations. A possible justification for this result could be associated with a higher intensity training in the beginning of the week and a consequent reduction as the next match approaches. However, Owen et al. [29] reported that, on MD-3, external TL variables were higher than on MD-(4, 2, 1). Moreover, Malone et al. [31] noted a progressive increase in total distance until MD-3 and a subsequent decrease until MD-1. A possible explanation for this difference could be that the study [29] was conducted during a competitive six-week mesocycle training period and that the other study [27] was conducted over the course of three separate weekly microcycles from the beginning, half and end of the season. 

Specifically, the major difference occurred on MD+1, and the results were significant despite the short training duration (~26 min) after home matches. For external TL, AvS was the second highest on MD+1. On the other hand, despite the short training duration (~16 min) after away matches, the AvS was the highest compared to the other days. A possible explanation for this result could be the need to compensate for the short training duration and, therefore, an increment in total distance covered, especially for non-starters [32,33]. As in previous studies, the inclusion criteria adopted for this study included players that completed at least 60 min in one match in the first half of the season and one match in the second half of the season, regardless of whether they were starters [32,33]. This could possibly explain a greater effort by non-starter players on the day after the match, along with the fact that four CDs, four WDs, five CMs and four WMs were included for analysis, but usually only two players from each position play. All these arguments may influence the data collected. 

Regardless of match location, the results for MD + 1 can be associated with a high-intensity training session. Moreover, it is important to acknowledge that an in-season match-day-minus training comparison was analyzed using mean values and that the microcycles/weeks have different patterns. For example, some microcycles had training days after match days and some did not. 

### 4.2. Comparison of Match Location

The rationale to compare TL data preceding home versus away matches is based on previous research [1,6], which has found evidence of multiple home advantage effects on technical, tactical and strategic behaviors in professional soccer. Thus, home matches increase ball possession compared to away matches [4,6]. Moreover, home teams tend to employ a more offensive strategy, performing a higher number of attacking actions (goals scored, shots on goal, passes, crosses and so on), while more defensive behaviors (interceptions, clearances, etc.) were evident in less advanced pitch positions when playing away [7,8]. Although these findings only regard data from matches, our study also observed some influences in training sessions due to match location. 

S-RPE remains similar during MD-(5, 4, 3, 2, 1) regardless of match location, but it is significantly higher on the day following a home match. In opposition to MD + 1, Abbott et al. [11] have found that s-RPE is similar for home and away matches. It is not clear why this is the case, because external TL was found to be significantly higher on the day following an away match. However, some studies have also reported that s-RPE did not reflect external TL [24,34]. Ferraz et al. [35] noted that RPE may be a physiological and volatile construct that could differ according to the cognitive focus of the player. 

Regarding MD-1, HI scores are in accordance with two studies [11,23]. Both stated that match location does not influence subjective wellbeing status. Moreover, our results are in line with other studies that determined there is no difference in player mood or stress between home and away matches [10,36]. 

For MD + 1, all scores from the HI were higher after an away match than a home match. These results are not corroborated by Abbott et al. [11], who found that sleep quality was lower when an away match was played. On the other hand, the stress values are in accordance with Abbott et al. [11], who also found higher values of stress if the match was played away vs. at home. In the days following an away match, our findings could be related to air travel [36], although this study is not in line with ours, because it reported that air travel had minimal influence on perceived fatigue, soreness, sleep quality and stress in six elite Australian soccer players 1 and 2 days after an away match. The authors found that soreness and stress were higher after home than away matches. Some explanations for these different findings could be associated with the methods used for data collection. Fowler et al. [36] measured these effects 2 days after the match and analyzed players from an elite professional squad in Australia.

Other factors that could affect stress on the day following an away match include travel, unfamiliarity with surroundings, habit disruption, changes in food provision, pressure from away supporters and sleep loss [37].

Furthermore, sleep quality was lower in the present study after away matches, which may be because the players went to sleep later and/or had to travel a further distance to get home, both of which could negatively influence perceived sleep quality [38]. 

Regarding external TL variables, for MD-5, it was observed that total distance and HSRD covered increased closer to an away match than a home match. For MD-4 and MD-3, external TL variables, such as total distance, HSRD and AvS, exhibited higher values nearer an away match than a home match. For MD-2, all external variables exhibited higher values closer to an away match than a home match. It is not clear why this happened, and the extant literature neither confirms nor denies our results. Thus, it appears that external TL is more intense between MD-5 and MD-2 as an away match approaches because, on MD-1, duration of training sessions and total distance covered are greater than when a home match approach. We speculate that this could be associated with not having to travel (such as in the case of an away match), in which case coaches apply more TL because they know that players will have time to recover. Moreover, the higher values between MD-5 and MD-2 when an away match approaches could be associated with more defensive behaviors (interceptions, clearances, and so on) [7,8]. Based on this knowledge, coaches try to apply a greater stimulus in a training session in order to achieve better results. 

For MD + 1, AvS was higher after an away than a home match, but duration of training sessions, total distance and HSRD were lower after an away than a home match. In general, the training session after a match has a lower duration. That fact can lead to a training session with exercises that achieve HSRD, but with a lower total distance covered. However, our study presents some differences regarding match location that we cannot address. 

**Figure 5:**
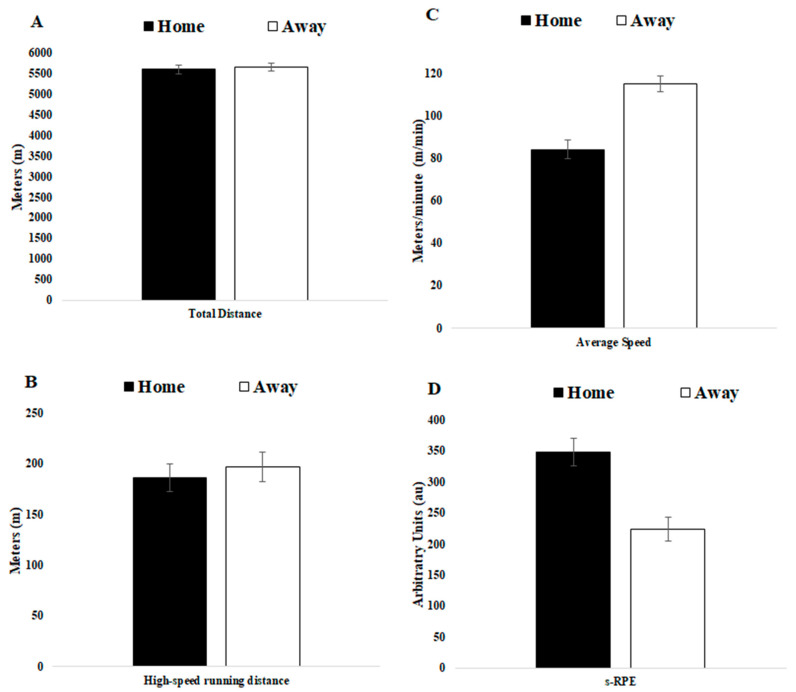
Average week training load regarding home and away matches for (**A**) total distance, (**B**) high-speed running distance, (**C**) average speed and (**D**) s-RPE.

**Figure 6:**
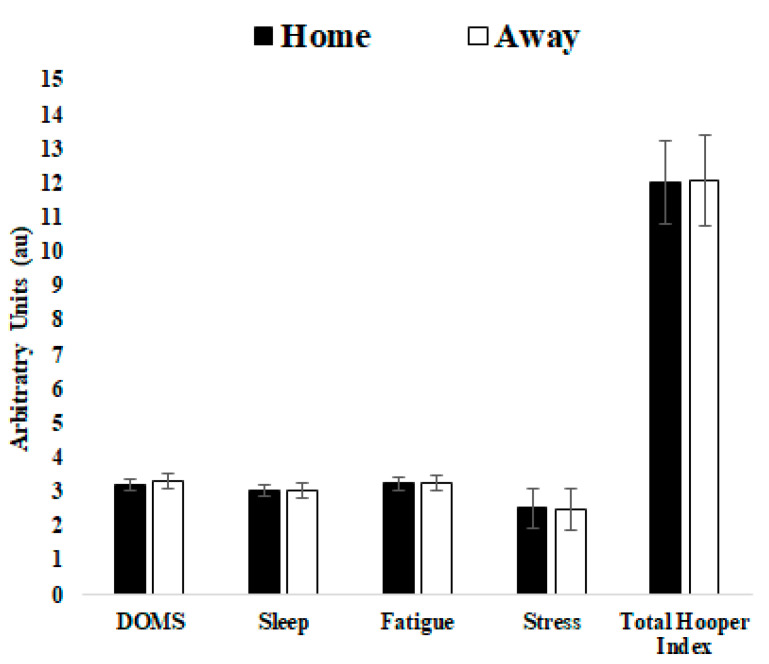
Average week training load regarding home and away matches for DOMS, sleep, fatigue, stress and total Hooper index.

Although it was not a purpose of this study, we have provided Figure 5 and Figure 6 with average week TL data regarding home and away matches as a tool to support coaches in their TL week planning when a home or away match approaches. 

Based on the statement of Barret et al. [39], there is a need for further investigation into what influences the results obtained by RPE to better understand how and if this helps inform practitioners of either mental or physical fatigue. For example, situations such as scoring a goal, opportunities to score a goal, interceptions, tackles, a good set play, a turnover win, increased possession or the ability to block an attack or even the non-technical/tactical training type of exercises may influence the perceived exertion of a player. In addition, HI scores can also be influenced in a similar manner. Nonetheless, this study reinforces the use of HI scores, especially on the day following a match. 

Furthermore, this study has some limitations that should be addressed. First, only one team was analyzed. Secondly, the team played in European matches and thus may not be representative of the customary training demands of other domestic teams that did not play in European matches [40] or those from different continents/countries [41] because the team manager and coach may have been influenced by different managerial and coaching philosophies [42].

Moreover, future research should consider different analyses of the season (e.g., to include weeks with two or three matches) and other contextual variables like match result [11,20,23] and level of opponent [11,23].

## 5. Conclusions

This study confirms and provides evidence regarding the match location influence on internal and external TL data preceding home and away matches from a team that played in European competitions. 

From a practical perspective, the findings of the present study can help to guide coaches for better TL periodization when a home or an away matches approaches for weeks with one match. For instance, when an away match approaches, it was shown that on MD-5, total distance and HSRD were higher. MD-4 showed higher values for all external variables. MD-3 showed higher values for total distance and AvS. Moreover, it was revealed that all HI scores and AvS were higher on MD + 1.

On the other hand, when a home match approaches, MD-1 showed higher values of total distance while MD + 1 showed higher values for s-RPE, total distance and HSRD.

## Figures and Tables

**Table 1:** Comparison of MD- for training load data that preceded home versus away matches for squad average, mean ± SD.

	Home	Away	ES (Home vs. Away)
**MD-5 (*n* = 24)**	***n* = 12**	***n* = 12**	
DOMS (au)	3.530 ± 0.239 ^a,b,c,d^	3.878 ± 0.256 ^a,b,c,d^	−1.41 (−2.07, −0.69) *
Sleep (au)	3.093 ± 0.142 ^c,d,e^	2.820 ± 0.166 ^e^	1.77 (1.01, 2.46)
Fatigue (au)	3.577 ± 0.245 ^a,c,d^	3.722 ± 0.246 ^a,b,c,d,e^	−0.59 (−1.21, 0.05)
Stress (au)	2.599 ± 0.0136	2.466 ± 0.181	0.83 (0.17, 1.46)
HI (au)	12.893 ± 0.557 ^c,d,e^	12.886 ± 0.651 ^a,b,c,d,e^	0.01 (−0.61, 0.63)
Duration (min)	56.642 ± 2.296 ^a,b,c,d,e^	57.807 ± 2.275 ^a,b,c,d,e^	−0.51 (−1.13, 0.13)
s-RPE (au)	190.658 ± 24.086 ^a,b,c^	180.746 ± 19.385 ^a,b,c,e^	0.45 (−0.18, 1.07)
Total Distance (m)	7050.871 ± 168.175 ^a,c,d,e^	7210.571 ± 120.153 ^a,c,d,e^	−1.09 (−1.73, −0.41)
HSRD (m)	254.122 ± 19.128 ^d,e^	316.044 ± 27.984 ^b,c,d,e^	−2.58 (−3.36, −1.70) *
AvS (m/min)	129.597 ± 6.448 ^a,b,c,d,e^	129.416 ± 5.051 ^a,b,c,d,e^	0.03 (−0.59, 0.65)
**MD−4 (*n* = 20)**	***n* = 10**	***n* = 10**	
DOMS (au)	2.848 ± 0.196 ^e^	2.824 ± 0.194 ^e^	0.12 (−0.50, 0.74)
Sleep (au)	2.984 ± 0.141 ^e^	2.927 ± 0.154 ^e^	0.39 (−0.25, 1.00)
Fatigue (au)	2.854 ± 0.185 ^e^	2.710 ± 0.193 ^c,e^	0.76 (0.11, 1.39)
Stress (au)	2.552 ± 0.176	2.471 ± 0.169	0.47 (−0.17, 1.09)
HI (au)	11.238 ± 0.530 ^c,d^	10.928 ± 0.575 ^e^	0.56 (−0.08, 1.18)
Duration (min)	81.083 ± 1.020 ^d,e^	77.346 ± 1.078 ^e^	3.56 (2.51, 4.47) *
s-RPE (au)	355.150 ± 25.845 ^d,e^	338.452 ± 27.881 ^d,e^	0.62 (−0.03, 1.24)
Total Distance (m)	6156.369 ± 94.723 ^b,c,d,e^	6519.533 ± 123.547 ^c,d,e^	−3.30 (−4.17, −2.29) *
HSRD (m)	252.113 ± 18.286 ^d,e^	273.032 ± 17.485 ^c,d,e^	−1.17 (−1.81, −0.48)
AvS (m/min)	76.034 ± 1.209 ^b,d^	84.475 ± 1.783 ^c,d,e^	−5.54 (−6.77, −4.09) **
**MD-3 (*n* = 24)**	***n* = 12**	***n* >= 12**	
DOMS (au)	2.929 ± 0.181 ^e^	2.822 ± 0.194 ^e^	0.57 (−0.07, 1.19)
Sleep (au)	3.011 ± 0.167 ^e^	2.919 ± 0.529 ^e^	0.23 (−0.39, 0.85)
Fatigue (au)	2.975 ± 0.191 ^e^	2.793 ± 0.208 ^e^	0.91 (0.24, 1.54)
Stress (au)	2.546 ± 0.144	2.253 ± 0.187 ^c^	1.76 (1.00, 2.45) **
HI (au)	11.461 ± 0.547 ^e^	10.786 ± 0.621 ^e^	1.15 (0.46, 1.80)
Duration (min)	80.978 ± 1.126 ^d,e^	78.534 ± 0.928 ^e^	2.37 (1.52, 3.12) *
s-RPE (au)	392.009 ± 22.746 ^c,d,e^	368.139 ± 30.510 ^d,e^	0.89 (0.22, 1.52)
Total Distance (m)	6643.648 ± 112.012 ^c,d,e^	6864.267 ± 65.982 ^c,d,e^	−2.40 (−3.16, −1.55) *
HSRD (m)	236.208 ± 13.133 ^d,e^	238.649 ± 15.622 ^d,e^	−0.17 (−0.79, 0.46)
AvS (m/min)	82.181 ± 1.287 ^d^	87.575 ± 1.169 ^c,d,e^	−4.39 (−5.43, −3.17) **
**MD-2 (*n* = 24)**	***n* = 12**	***n* = 12**	
DOMS (au)	2.980 ± 0.203 ^e^	3.079 ± 0.191 ^e^	−0.50 (−1.12, 0.14)
Sleep (au)	2.672 ± 0.163 ^e^	2.787 ± 0.149 ^e^	−0.74 (−1.36, −0.08) *
Fatigue (au)	2.942 ± 0.217 ^e^	3.090 ± 0.193 ^e^	−0.72 (−1.35, −0.07) *
Stress (au)	2.475 ± 0.160	2.438 ± 0.165	0.23 (−0.40, 0.84)
HI (au)	11.111 ± 0.608 ^e^	11.393 ± 0.553 ^e^	−0.49 (−1.10, 0.15)
Duration (min)	77.704 ± 0.684 ^d,e^	78.933 ± 0.477 ^e^	−2.08 (−2.81, −1.28)
s-RPE (au)	309.385 ± 22.746 ^c,d,e^	319.927 ± 23.016 ^d,e^	−0.46 (−1.08, 0.18)
Total Distance (m)	5672.056 ± 66.924 ^d,e^	5772.040 ± 57.580 ^d,e^	−1.60 (−2.28, −0.86)
HSRD (m)	202.866 ± 9.509 ^d,e^	208.496 ± 12.475 ^d,e^	−0.51 (−1.13, 0.13)
AvS (m/min)	73.035 ± 1.041 ^d^	73.167 ± 0.841 ^d,e^	−0.14 (−0.76, 0.48)
**MD-1 (*n* = 24)**	***n* = 12**	***n* = 12**	
DOMS (au)	2.914 ± 0.170 ^e^	2.834 ± 0.220 ^e^	0.41 (−0.23, 1.02)
Sleep (au)	2.601 ± 0.148 ^e^	2.713 ± 0.164 ^e^	−0.72 (−1.34, −0.06)
Fatigue (au)	2.887 ± 0.185 ^e^	2.828 ± 0.222 ^e^	0.29 (−0.34, 0.91)
Stress (au)	2.398 ± 0.150	2.515 ± 0.197	−0.67 (−1.29, −0.02)
HI (au)	10.801 ± 0.512 ^e^	10.889 ± 0.619 ^e^	−0.15 (−0.77, 0.47)
Duration (min)	86.379 ± 0.651 ^e^	82.954 ± 1.303 ^e^	3.33 (2.31, 4.20) *
s-RPE (au)	218.543 ± 15.538 ^e^	221.074 ± 13.389 ^e^	−0.17 (−0.79, 0.45)
Total Distance (m)	3644.602 ± 62.053 ^e^	3452.107 ± 66.846 ^e^	2.98 (2.03, 3.82) **
HSRD (m)	69.503 ± 6.994	68.431 ± 5.338	0.17 (−0.45, 0.79)
AvS (m/min)	42.245 ± 0.775 ^e^	41.877 ± 1.044 ^e^	0.40 (−0.23, 1.02)
**MD + 1 (*n* = 20)**	***n* = 10**	***n* = 10**	
DOMS (au)	4.048 ± 0.265	4.377 ± 0.267	−1.24 (−1.89, −0.54) *
Sleep (au)	3.737 ± 0.156	4.005 ± 0.230	−1.36 (−2.02, −0.65)
Fatigue (au)	4.158 ± 0.250	4.444 ± 0.250	−1.14 (−1.79, −0.45) *
Stress (au)	2.526 ± 0.179	2.687 ± 0.201	−0.85 (−1.47, −0.18)
HI (au)	14.469 ± 0.684	15.513 ± 0.699	−1.51 (−2.18, −0.78) *
Duration (min)	26.687 ± 3.098	16.179 ± 0.769	4.66 (3.39, 5.74) **
s-RPE (au)	86.238 ± 23.532	25.922 ± 2.432	3.61 (2.54, 4.53) *
Total Distance (m)	4421.407 ± 114.412	4308.190 ± 82.567	1.13 (0.45, 1.78)
HSRD (m)	103.066 ± 16.503	77.741 ± 8.651	1.92 (1.14, 2.63)
AvS (m/min)	102.210 ± 16.029	273.645 ± 11.738	−12.20 (−14.65, −9.27) **

MD- = match day minus (5, 4, 3, 2, 1); MD + 1 = match day plus 1; DOMS = delayed onset muscle soreness; au = arbitrary units; HI = Hooper Index; min = minutes; m = meters; s-RPE = session rating of perceived exertion; HSRD = high-speed running distance; AvS = average speed; ES = effect size. ^a^ denotes difference from MD-4. ^b^ denotes difference from MD-3. ^c^ denotes difference from MD-2. ^d^ denotes difference from MD-1. ^e^ denotes difference from MD + 1. All, *p* < 0.05. * significant differences between home vs. away (*p* < 0.05). ** significant differences between home vs. away (*p* < 0.01).

**Table 2:** Comparison of TL between starters and non-starters for home and away matches on MD-5 and MD + 1; mean ± SD.

MD-5	Home (Starter, *n* = 10)	Home (Non-Starter, *n* = 10)	Away (Starter, *n* = 10)	Away (Non-Starter, *n* = 10)
DOMS (au)	3.907 ± 0.990	3.236 ± 0.934	4.052 ± 0.997	3.739 ± 1.248
Sleep (au)	3.148 ± 0.666	3.035 ± 0.751	2.991 ± 0.578	2.701 ± 0.778
Fatigue (au)	3.909 ± 0.988	3.227 ± 0.903	4.038 ± 0.918	3.448 ± 1.129
Stress (au)	2.485 ± 0.966	2.666 ± 0.879	2.387 ± 0.873	2.577 ± 1.035
HI (au)	13.449 ± 2.552	12.308 ± 2.594	13.468 ± 2.351	12.465 ± 3.526
Duration (min)	52.599 ± 12.598	60.536 ± 7.727	54.550 ± 4.946	62.228 ± 14.944
s-RPE (au)	216.723 ± 143.317	171.020 ± 36.071	161.122 ± 55.633	212.840 ± 132.008
Total Distance (m)	7242.072 ± 526.893	6811.405 ± 997.150	7258.627 ± 619.855	7203.919 ± 411.725
HSRD (m)	226.664 ± 114.584	271.157 ± 165.186	338.507 ± 136.427	306.065 ± 149.409
AvS (m/min)	144.588 ± 33.356	114.324 ± 23.714	134.108 ± 14.039	122.215 ± 31.009
**MD + 1**				
DOMS (au)	4.621 ± 0.991 ^a^	3.513 ± 0.911	4.664 ± 1.013	4.145 ± 1.313
Sleep (au)	3.950 ± 0.652	3.539 ± 0.971	3.800 ± 0.788	4.207 ± 1.174
Fatigue (au)	4.625 ± 0.917 ^a^	3.685 ± 0.868	4.469 ± 0.962	4.193 ± 1.012
Stress (au)	2.733 ± 0.919	2.354 ± 0.967	2.602 ± 1.065	2.798 ± 1.180
HI (au)	15.930 ± 2.242 ^a^	13.087 ± 3.089	15.753 ± 2.487	15.343 ± 4.009
Duration (min)	21.685 ± 8.076	30.543 ± 20.128	16.725 ± 3.854	16.075 ± 3.399
s-RPE (au)	46.111 ± 34.241	122.407 ± 151.091	29.309 ± 12.157	23.254 ± 10.022
Total Distance (m)	4215.586 ± 258.536	4649.747 ± 678.602	4435.604 ± 341.846	4260.829 ± 144.671
HSRD (m)	66.486 ± 29.941	139.718 ± 113.176	75.039 ± 40.146	84.345 ± 58.732
AvS (m/min)	140.265 ± 89.796	74.532 ± 46.093	275.079 ± 53.178	272.742 ± 54.309

MD-5 = match day minus 5; MD + 1 = match day plus 1; DOMS = delayed onset muscle soreness; au = arbitrary units; HI = Hooper index; min = minutes; m = meters; s-RPE = session rating of perceived exertion; HSRD = high-speed running distance; AvS = average speed. ^a^, denotes difference from home (non-starter), all *p* < 0.05.

## Data Availability

The data presented in this study are available on request from the corresponding author.
